# Nocturia is associated with stiffer central artery and more likely development of major adverse cardiovascular events in men

**DOI:** 10.3389/fruro.2023.1113054

**Published:** 2023-01-26

**Authors:** C. K. Chan, Chi Fai Ng, Steffi K. K. Yuen, B. S. Y. Lau, C. H. Yee, J. Y. C. Teoh, P. K. F. Chiu, S. W. Kwok

**Affiliations:** ^1^ SH Ho Urology Centre, Department of Surgery, The Chinese University of Hong Kong, Hong Kong, China; ^2^ Department of Surgery, Prince of Wales Hospital, Shatin, Hong Kong, China

**Keywords:** central arterial stiffness, Lower urinary tract symptom (LUTS), major adverse cardiovascular events (MACE), nocturia, pulse wave velocity

## Abstract

**Objectives:**

To study the association between nocturia and brachial-ankle pulse wave velocity (baPWV) [surrogate of central arterial stiffness (CAS)] in men and to explore this association on predicting major adverse cardiovascular events (MACE).

**Methods:**

246 consecutive men (mean age:68.1 ± 8.4, range 36-88) newly referred to urology clinic for male-lower urinary tract symptoms(mLUTS)/nocturia were recruited. Their bilateral baPWV were measured by automatic pulse waveform analyzer. The associations between baseline characteristics, mLUTS/nocturia and baPWV(>1800cm/sec) [significant CAS] were analyzed by multivariate logistic regression. We followed the cohort for a median period of 27.5 months. Cox proportional hazard regression analysis and Kaplan-Meier method were used to study factors predicting MACE.

**Results:**

The mean ( ± SE) baPWV of our cohort was 1820 ± 16cm/sec. For comparison, the reported value of the general population of similar age structure was~1650cm/sec. IPSS (total) was not associated with baPWV, whereas IPSS-Question.7(nocturia) was significantly increased with baPWV in men<70yo [nocturia=1.6 ± 1.14, 2.1 ± 1.08, 2.67 ± 1.33) for baPWV(cm/sec) <1400, 1400-1800, >1800 respectively] (P-trend=0.002). Age≥70yo (OR:2.70, 95%CI:1.52-4.76), diabetes mellitus (OR:2.26, 95%CI:1.06-4.83), hypertension (OR:1.95, 95%CI:1.10-3.45) and nocturia≥3x/night (OR:1.75, 95%CI:1.02-3.12) independently determined baPWV>1800cm/sec. The cumulative incidence rate of MACE was 46.8/1000 man-years(95%CI:30.96-68.16/1000). The addition of nocturia≥3x/night and baPWV>1800 cm/sec to the basic model improved the prediction of the development of MACE (difference in -2 log likelihood value: 11.219, p<0.001). Past history of ischemic heart (HR:5.67, 95%CI:2.02-15.88), nocturia≥3x/night (HR:2.87, 95%CI: 0.94-8.76) and baPWV>1800cm/sec (HR:5.16, 95%CI:1.79-14.90) independently predicted MACE in men.

**Conclusion:**

Men attending the urology clinic for male-LUTS/nocturia had higher baPWV. This association was more pronounced in men<70yo. Men presented with both nocturia≥3x/night and baPWV>1800cm/sec showed significant predilection for developing MACE.

## 1 Introduction

### 1.1 Background & rationale

Nocturia was considered the most bothersome entity amongst lower urinary tract symptoms in men (mLUTS) (1). Besides, particularly in men younger than 60 years of age, it was also associated with vascular disorders like hypertension(HT) of young onset (2), impaired control of nocturnal blood pressure(BP) (3), coronary artery disease(CAD) and possibly increased incidence of major adverse cardiovascular events(MACE) [namely, acute myocardial infarction(AMI), ischemic heart disease(IHD), heart failure, coronary angioplasty, stroke(CVA), transient ischemic attack(TIA), intracerebral hemorrhage, cerebrovascular disease and peripheral vascular disease(PVD)] (4).

Central arterial stiffness(CAS) is one of the earliest detectable functional and structural changes in the vascular wall, preceding the clinical presentation of coronary artery disease(CAD) and hypertensive heart disease (5). CAS is increased with age even in people without much atherosclerosis (arteriosclerosis) (6) and can be quantified simply by the measuring the aortic pulse wave velocity(PWV) (7). Brachial-ankle PWV (baPWV) is linearly related to aortic PWV and its measurement has become a surrogate of CAS due to its non-invasiveness, ease of usage, reproducibility, wide acceptability by the patients and strong prediction for the future MACE (8).

However, the inter-relationship between self-reported nocturia, CAS and the future MACE in men is not well established and remains unclear. Herein, this study was to report (1) the cross-sectional correlation between baPWV and self-reported nocturia in men, (2) and then the longitudinal observation of the same cohort in an effort to determining the impact of both parameters on predicting the development of MACE in men.

## 2 Methods

### 2.1 Objectives

The objectives of the study included:

(a) To study the relationship between baPWV and nocturia in male patients
(b) To study the effects of baPWV and nocturia on the development of major adverse cardiovascular events (MACE) in future

### 2.2 Study design

This study was a single cohort longitudinal study, characterized by two phases. The first phase was a cross-sectional study reporting the association(s) among baPWV, self-reported nocturia and other baseline clinical characteristics, whereas the second phase was a longitudinal observational study of the same study cohort for the development of MACE and the factors predicting its occurrence. This study was approved by the ethical committee of the institute (CREC Ref No.: 2019.400) and executed in accordance with the Good Clinical Practice guidelines. Informed consent was obtained from all study subjects.

### 2.3 Setting

The study was performed in a tertiary urological referral centre for male lower urinary tract symptoms (mLUTS), from 1^st^ September 2017 to 31^st^ May 2018. During the initial patient assessment, a series of examination, including both urological and cardiovascular assessment (for the promotion of men health) were performed. The patients would then regularly follow-up in our hospital for their voiding situation. Furthermore, territory-wide electronic hospital record was used in all public hospitals in our region. Therefore, additional follow-up information, including the development of MACE, would be retrieved from the system for subsequent analysis.

### 2.4 Participants

We recruited men ≥ 18years old (yo) who consecutively attended the general urology clinic for mLUTS/nocturia for the study. Those men having history of neuropathic bladder, prostatic surgery, prostate cancer, active urinary tract infection and untreated urethral strictures were excluded. In view of the measurement method of the baPWV device (oscillometric method), those men having history of aortic aneurysm, marked cardiac arrhythmia and severe peripheral vascular disease were also excluded for study.

### 2.5 Variables

In this study, variables collected for analysis included (a) urological variables - International Prostate Symptoms Score (IPSS), nocturia episodes (as from question 7 of IPSS), International Index of Erectile Function (IIEF-5) score, maximal uroflow rate and postvoid residual urine; (b) Cardiovascular and metabolic variables – the presence of common cardiovascular conditions, total cardiovascular prediction score, blood sugar and lipid levels, baseline blood pressure and basal metabolic index (BMI); (c) Variables related to vascular functions - Brachial-ankle PWV (baPWV) and Ankle-Brachial Index (ABI). The details of individual variables measurement would be described in subsequent section.

### 2.6 Data sources and measurements

#### 2.6.1 Urological variables

Upon consultation for male LUTS, the male subjects filled out both International Prostate Symptoms Score (IPSS) and International Index of Erectile Function (IIEF-5) questionnaires. In this study, the term nocturia was referred to its original definition published in 2002: “The complaint that the individual has to wake at night one or more times to void” (9). The self-reported nocturia episodes were based on the score of IPSS Q.7 (addressing nocturia). They would also undergo uroflowmetry/post-void residual urine estimation by the bladder scan.

#### 2.6.2 Cardiovascular and metabolic variables

Subjects were also evaluated for the smoking status, history of HT, diabetes mellitus (DM), CVA, and IHD, which were cross-checked with the diagnostic codes of the central registry and medical records. Blood glucose, total lipoprotein, high density lipoprotein (HDL), and renal function were checked. The general and composite cardiovascular disease prediction score [total CVD scores], as reported by D’Agostino (10), was determined for each individual subject. In brief, the CVD scoring system by D’Agostino is a prediction tool to assess a 10-year estimate risk of developing general cardiovascular disease (CVD) [i.e. coronary heart disease, cerebrovascular disease, peripheral vascular disease and heart failure], rather than just the risk of a single component of CVD, based on age, smoking status, total and HDL cholesterol, systolic BP (sBP), treatment for HT and DM of each individual. The points scored by an individual can be inferred to his heart age/vascular age of an individual without risk factors. The possible range varied from -2 to +33: the higher the value, the greater the cardiovascular risk.

#### 2.6.3 Vascular function variables

The assessment of the central artery stiffness (CAS) was incorporated into the baseline urological evaluation for part of a pilot men’s health programme. Upon the measurement of CAS, the subjects were instructed to rest in supine position for 10 minutes followed by measurement of their bilateral baPWV and Ankle-Brachial Index (ABI) with the use of the automatic device (pulse waveform analyzer, model VP 1000 Plus PWV/ABI: Omron-Colin Co., Tokyo, Japan) (**Supplementary Figure 1**). The device adopted the oscillometric method to record baPWV and blood pressure. In brief, baPWV was measured simply by wrapping the blood pressure cuffs on the four extremities and calculated as the ratio of the virtual arterial path length(cm) derived from the height of the men and the time difference(sec) between the commencements of systolic increases in brachial and ankle pressure waves. The test was repeated thrice and the mean value of the measurements was taken as the final reading.

#### 2.6.4 Follow up evaluation/Acquisition of cardiovascular events

MACE were monitored across the follow-up period with the utilization of our territory-wide electronic patient record system based on diagnostic codes of 10^th^ edition of International Statistical Classification of Diseases and Related Health Problems (ICD-10), as reported by us in an earlier study (11), and hospital medical records locally and in other hospitals. Diagnostic codes used for MACEs include angina pectoris (I20), acute myocardial infarction [AMI] (I21), subsequent myocardial infarction (I22), complications following AMI (I23), other acute ischaemic heart disease (I24), chronic ischaemic heart disease (I25), heart failure (I50), coronary angioplasty (Z95)/coronary artery bypass surgery (Z98), transient cerebral ischaemic attack (G45, G46), stroke (I65. I66, I67), cerebrovascular diseases (I61, I62, I63, I64 – I67), atherosclerosis of aorta/extremities (I70), and peripheral vascular diseases (PVD) (I73, I74).

### 2.7 Bias

We enrolled male subjects consecutively at the urological centre. The use of the territory-wide computerized electronic medical record tracking system was to lower the chance of losses to follow-up and able to retrieve relevant information as to whether the subject developed MACE across the observation period, and hence to keep the differential mis-classification of the outcome to the minimum. Researcher involved in the retrieval of follow-up information was blinded from the baseline clinical parameters to minimizing bias on data collection.

### 2.8 Study sample size

Previous studies showed that baPWV was correlated with age for R~0.4-0.6 (12). We hypothesized that baPWV was correlated with mLUTS/nocturia for R~0.16-0.20. Sample size calculation showed that ~240 subjects (α=0.05; β=0.80) had to be recruited for the study.

### 2.9 Quantitative variables handling

Patients were divided into younger age (<70 yo) and older group (70 yo or above) based on the cut-off at the median age of 69.3 yo. For IPSS, patients were divided into mild to moderate symptoms (total score < 20) and severe symptom (total score 20 or above). For other baseline continuous variables, including BMI, peak flow rate, etc. were dichotomized based on their median values. The dichotomized variables were then, together with baseline categorical variables, used as co-variates and analyzed by multivariate logistic regression.

### 2.10 Statistical methods

Continuous data with normal distribution were presented as means ± standard deviation (SD), medians(range) for those with skewed distribution; categorical data as proportions/percentages. Pearson tests for bivariate correlation coefficients among the continuous variables were performed. Baseline data were compared across age (<70yo and ≥70yo) and nocturia (≤2x/night vs ≥3x/night) categories, by using the χ^2^ test/Fisher-Exact test for categorical data, the unpaired Student *t* test for data of 2 groups, parametric ANOVA test for multiple groups for continuous data with normal distribution, Mann-Whitney U test for skewed data of 2 groups/Kruskall-Wallis test for skewed data of multiple groups, where appropriate.

Baseline continuous variables were dichotomized based on their median values. The dichotomized variables were then, together with baseline categorical variables, used as co-variates and analyzed by multivariate logistic regression using stepwise backward LR method to study the factor(s) [age, BMI, waist circumference, smoking status, IPSS(total), IIEF-5, nocturia based on Q.7 of IPSS, HT, DM, prior IHD, prior CVA, total CVD scores>15, Qmax, voided volume, PVR, ABI] determining baPWV>1800 cm/sec, a cut-off value highly associated with development of MACE (e.g. acute coronary syndrome) (13). Any variables for which probability (p) identified by univariate analysis as <0.1 were also included in the multiple logistic regression analysis.

Time to MACE was modelled with nocturia and baPWV after adjustment for age, BMI, waist circumference, smoking status, IIEF, IPSS(total), history of HT, DM, CVA, IHD, heart disease and total CVD scores>15 (10) by using Cox proportional hazard regression analysis. The incremental values of nocturia and baPWV were assessed using a modified stepwise procedure in 3 modeling steps. In Model 1, age, BMI, waist circumference, smoking status, IIEF-5, IPSS (total), HT, DM, prior history of CVA, IHD and total CVD scores>15, were entered into the model as co-variates, whereas nocturia was added to Model 2 together with Model 1 covariates and baPWV>1800cm/sec plus Model 2 co-variates were added to Model 3. An improvement in model prediction was based on the −2 log likelihood ratio statistic, which was based on a difference in the −2 log likelihood value; and the p-value was based on the incremental value compared with the previous model.

The survival curves for MACE were depicted by the Kaplan-Meier method. The log-rank test was used to compare the groups for the time‐to‐event end point analyses. Statistical analysis was performed using IBM SPSS-V.24 statistical package. Statistical significance was taken when p<0.05.

## 3 Results

### 3.1 Participants

From 1^st^ September 2017 to 31^st^ May 2018, 246 men (mean age: 68.13 years, SD:8.41, range:36–88) were recruited.

### 3.2 Cross-sectional observation

#### 3.2.1 Descriptive data

The mean ( ± SD) value of left baPWV(cm/sec) and nocturia were 1821.45 ± 388.57 and 2.65 ± 1.32 respectively. The left baPWV was highly correlated with that on the right (R^2 =^ 0.868, p<0.001) and was taken as the baPWV reading of the individual subject (**Supplementary Figure 2**). The men were stratified into 4 groups according to their median age (<70 vs ≥70yo) and median episodes of self-reported nocturia (≤2x/night vs ≥3x/night). The comparison of the baseline demographic and clinical characteristics of the subjects was tabulated in **Table 1**.

**Table 1:** Baseline demographic data and characteristics of the whole cohort and the respective groups stratified by age (<70 years old vs ≥70 years old) and nocturia episodes (≤2x/night vs ≥3x/night).

Variables	Whole cohort N=246	Age < 70		Age ≥ 70	
		Nocturia ≤ 2	Nocturia ≥ 3	Nocturia ≤ 2	Nocturia ≥ 3
		(N=83) Ref.	(N=55)	(N=38)	(N=70)
**Age[yr]**	68.13 (8.41)	61.35 (6.02)	63.45* (4.10)	74.44*** (4.30)	76.41*** (4.57)
**Height[m]**	1.65 (0.066)	1.67 (0.062)	1.66 (0.061)	1.63** (0.064)	1.64** (0.067)
**Weight[kg]**	65.4 (44.50-123.30)	67 (47.50–123.30)	67.5 (44.50–115.20)	64.5 (45.4–82.50)	63.1* (47.10–93.00)
**BMI[kg/m^2^]**	24.34 (3.28)	24.28 (3.54)	25.13 (3.38)	24.29 (3.41)	23.82 (2.73)
**Waist Circumference[cm]**	88.20 (8.76)	87.07 (8.73)	89.05 (9.40)	88.45 (8.90)	88.71 (8.27)
**IIEF-5**	3.00 (0–25)	5 (1–25)	4* (1–25)	3** (1–24)	1*** (0–20)
**IPSS[total]**	12.43 (7.44)	9.65 (6.48)	15.53*** (7.38)	9.05 (5.60)	15.14*** (7.51)
**Nocturia (IPSSQ.7)**	2.65 (1.32)	1.47 (0.61)	3.55*** (0.72)	1.61 (0.50)	3.91*** (0.81)
**Current Smoker**	27 (11.0%)	11 (13.3%)	7 (12.7%)	1 (2.6%)	8 (11.4%)
**DM**	43 (17.5%)	18 (21.7%)	6 (10.9%)	7 (18.4%)	12 (17.1%)
**HT**	134 (54.5%)	35 (42.2%)	28 (50.9%)	28** (73.7%)	43* (61.4%)
**CVA**	8 (32.5%)	2 (2.4%)	2 (3.6%)	0 (0%)	4 (5.7%)
**IHD**	37 (15.0%)	7 (8.4%)	6 (10.9%)	9* (23.7%)	16* (22.9%)
**Fasting glucose (mmol/L)**	5.70 (0.96)	5.73 (1.08)	5.68 (0.80)	5.74 (0.93)	5.66 (0.95)
**HDL** **(mmol/L)**	1.46 (0.42)	1.46 (0.43)	1.43 (0.40)	1.45 (0.50)	1.48 (0.38)
**LDL** **(mmol/L)**	2.62 (0.94)	2.65 (0.97)	2.84 (1.08)	2.62 (0.87)	2.41 (0.76)
**Total cholesterol (mmol/L)**	4.64 (1.09)	4.71 (1.10)	4.83 (1.22)	4.63 (1.00)	4.42 (0.97)
**Triglyceride (mmol/L)**	1.26 (0.68)	1.29 (0.60)	1.38 (0.91)	1.22 (0.61)	1.16 (0.60)
**Total CVD Score** **(2008) (range: -2 –+33)**	15.66 (3.73)	13.86 (3.93)	14.65 (3.18)	17.82*** (2.52)	17.34*** (3.12)
**Peak uroflow [ml/sec]**	12.56 (6.28)	13.88 (7.00)	13.24 (6.94)	11.54** (4.52)	10.98** (5.24)
**Voided Volume[ml]**	246.38 (137.30)	316.47 (163.19)	226.60*** (111.81)	229.70** (103.46)	187.64*** (98.90)
**PVR [ml]**	49 (0 – 496)	48 (0 – 456)	57.0 (0 – 385)	58.0 (0 – 496)	46.5 (0 – 428)
**Left ABI**	1.12 (0.12)	1.17 (0.096)	1.21* (0.10)	1.17 (0.10)	1.13 (0.15)
**Left baPWV[cm/sec]**	1821.45 (388.57)	1669.63 (287.41)	1789.36* (341.15)	1962.29*** (460.58)	1950.21*** (420.50)

continuous data are expressed as mean (SD) or median (range); categorical data are expressed as N (%); Ref, reference group.

(*) = p<0.05 ; (**) = p<0.01 ; (***) = p<0.001.

BMI, Body Mass Index; IIEF-5, International Index of Erectile Function; IPSS, International Prostate Symptom Score; Nocturia IPSS(Q.7), Question 7 of IPSS (Nocturia); DM, Diabetes Mellitus; HT, Hypertension; CVA, History of Cerebrovascular Accident / Stroke; IHD, History of Ischemic Heart Disease; Total CVD Score (2008): Cardiovascular Disease Prediction Score (2008) – a composite score (range: -2 to +33) taking into account the Age / High Density Lipoprotein (HDL) level / Total Cholesterol (TC) level / Systolic Blood Pressure (sBP) / Smoking Status / History of Diabetes Mellitus (DM) [10]; Peak uroflow:maximum micturition flow rate at the time of uroflowmetry PVR, Post-void Residual Volume; ABI, Ankle-Brachial Index; baPWV, Brachial-Ankle Pulse Wave Velocity.

#### 3.2.2 Outcome data

Of all the variables listed, baPWV was positively correlated with the age (R:0.370, p<0.0001), the total CVD scores (R:0.232, p<0.001) and the number of nocturia per night (R:0.170, p<0.01) [more pronounced for men <70yo, R: 0.20, p=0.017, see supplementary Fig 3.]; whereas it was negatively correlated with the weight (R:-0.150, p<0.01), the height (R:-0.240, p<0.0001), IIEF-5(R:-0.170, p< 0.01) and the voided volume (R:-0.200, p<0.01) respectively.

Compared with the reference group (age <70yo and nocturia ≤ 2x/night), subjects who were older (≥70yo) and having more severe nocturia (≥3x/night) got worse erectile function and LUTS, higher prevalence of HT, IHD, greater total CVD scores, slower micturition flow, lower voided volume for the uroflowmetry, lower ABI index and higher baPWV values (**Table 1**).

#### 3.2.3 Main result

**Figure 1:**
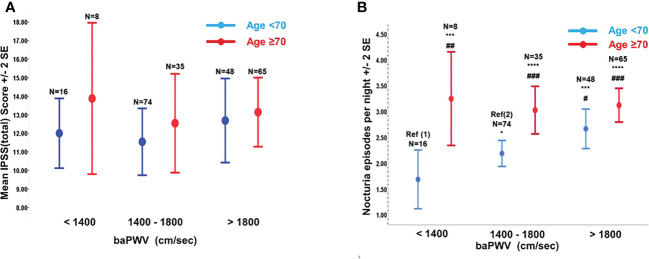
The variations of total International Prostate Symptoms Score [IPSS(total)] and nocturia episodes with brachial-ankle pulse wave velocity (baPWV). **(A)** Variation of IPSS(total) with baPWV (cm/sec) stratified by Age <70yr or Age ≥70yr. **(B)** Variation of Nocturia episodes with baPWV stratified by Age <70yr or Age ≥70yr. Compared to Ref (1): *p=0.090; ***p<0.010; ****p<0.001. Compared to Ref (2): #p<0.050; ##p<0.010; ###p<0.001.

While IPSS (total) was not associated with the age and baPWV in our cohort [**Figure 1A**], degree of self-reported nocturia was significantly increased with the baPWV when the subjects were <70yo [nocturia=1.6 ± 1.14, 2.1 ± 1.08(p=0.09), 2.67 ± 1.33 (p=0.031) for baPWV(cm/sec) <1400, 1400-1800, >1800 respectively] (P-trend = 0.002). Similarly, subjects<70yo with nocturia≥3x/night had significantly higher baPWV(cm/sec) [1789.36 ± 341.15 (nocturia≥3x/night) vs 1669.63 ± 287.41 (nocturia ≤ 2x/night), p<0.05]. Although subjects≥70yo had higher baPWV (1717.35 ± 314.29 vs 1954.46 ± 432.90, p<0.001) and reported more frequent nocturia (3.10 ± 1.32 vs 2.30 ± 1.21, p<0.001) than did those <70yo, no significant association was shown between baPWV and nocturia in this subgroup of subjects [Fig 1(b)].

Multivariate step-wise logistic regression analysis (**Table 2**) showed that age≥70yo (OR: 2.02, 95%CI: 1.10-3.70), nocturia ≥3x/night (OR:1.80, 95%CI:1.03-3.16), history of DM (OR:2.08, 95%CI: 0.97-4.48), history of CVA (OR:9.78, 95%CI: 1.07 – 89.60) and total CVD score>15 (OR:1.89, 95%CI:1.03–3.48) were the independent factors determining baPWV>1800cm/sec in our cohort. Based on the calculated CVD score at baseline, the vascular age of our cohort is ~6 years older than their chronological age. The corresponding vascular age of the subgroups of our cohort is shown in **Supplementary Table 1**.

**Table 2:** Multivariate logistic regression analysis of factors determining left-baPWV ≥ 1800 cm/sec.

		Univariate Analysis			Multivariate Analysis		
Independent Variable	N(%)	Odds ratio	95% CI	p-value	Odds ratio	95% CI	p-value
**Age ≥ 70yr.**	108(43.9)	2.834	1.684 –4.771	<0.001	2.020	1.102 – 3.702	0.023*
**BMI (kg/m^2^)≥ 25**	97(39.4)	0.783	0.468 – 1.311	0.352	NA	NA	NA
**Waistcircumference > 89 cm**	116(47.2)	1.413	0.854 –2.338	0.179	NA	NA	NA
**Current Smoker**	27(11.0)	1.294	0.581 – 2.883	0.527	NA	NA	NA
**IIEF-5 ≤ 7**	72(29.3)	1.790	1.016 – 3.151	0.044	1.096	0.581 – 2.068	0.777
**IPSS ≥ 20**	46(18.7)	1.098	0.578 – 2.086	0.775	NA	NA	NA
**Nocturia ≥ 3**	125(50.8)	2.015	1.210 – 3.354	0.007	1.800	1.027 – 3.155	0.040*
**DM**	43(17.5)	2.296	1.165 – 4.523	0.016	2.084	0.970 –4.477	0.060*
**HT**	134(54.5)	2.011	1.204 – 3.359	0.008	1.346	0.740 –2.448	0.330
**CVA**	8(32.5)	8.717	1.056 – 71.959	0.044	9.777	1.067 – 89.580	0.044*
**IHD**	37(15.0)	0.944	0.471 – 1.893	0.872	NA	NA	NA
**Total CVD (2008) >15**	126(51.2)	2.686	1.597 – 4.518	<0.001	1.892	1.028 – 3.481	0.040*
**(range: -2 – +33)**							
**Peak Uroflow<13ml/sec**	144(58.5)	1.329	0.795 –2.220	0.278	NA	NA	NA
**Voided volume**	151	1.633	0.967 –	0.067	1.302	0.712 –	0.391
**<250ml**	(61.4)		2.757			2.379	
**PVR < 90ml**	81(32.9)	0.745	0.437 – 1.270	0.280	NA	NA	NA
**Left ABI <0.9or >1.5**	6(2.4)	0.229	0.026 – 1.986	0.181	NA	NA	NA

NA, Not Applicable. (*) = p<0.05. BMI, Body Mass Index; IIEF-5, International Index of Erectile Function; IPSS, International Prostate Symptom Score; Nocturia, Question 7 of IPSS (Nocturia); DM, Diabetes Mellitus; HT, Hypertension; CVA, History of Cerebrovascular Accident/Stroke; IHD, History of Ischemic Heart Disease; Total CVD (2008), Cardiovascular Disease Prediction Score (2008) – a composite score (range: -2 to +33) taking into account the Age/High Density Lipoprotein (HDL) level/Total Cholesterol (TC) level/Systolic Blood Pressure (sBP)/Smoking Status/History of Diabetes Mellitus (DM) (10); Peak uroflow, maximum micturition flow rate at the time of uroflowmetry PVR, Post-void Residual Volume; ABI, Ankle-Brachial Index; baPWV, Brachial-Ankle Pulse Wave Velocity.

### 3.3 Longitudinal observation

#### 3.3.1 Descriptive data

Twenty-five incident cardiovascular events, namely heart failure(n=4), need of coronary angioplasty for asymptomatic coronary artery disease(n=6), CVA or TIA(n=5), unstable angina(n=9) and PVD(n=1), were reported across the median follow-up of 27.50 months (range:0.82-30.74). The cumulative incidence rate of the MACEs was 3.90/1000 man-months (95%CI:2.58–5.68/1000) or 46.8/1000 man-years (95%CI: 30.96-68.16/1000).

#### 3.3.2 Outcome data and main result

**Figure 2:**
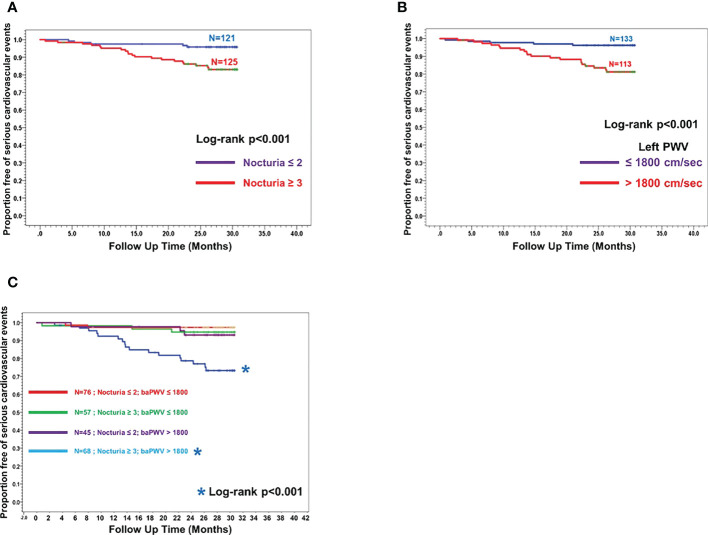
Kaplan Meier curves showing proportion of subjects free of major cardiovascular events (MACE-free) across the follow-up since the first measurement of baPWV. **(A)** Two sub-groups stratified by Nocturia≥3/night across the follow-up time (months) since the first baPWV measurement. **(B)** Two sub-groups stratified by baPWV>1800cm/sec across the follow-up time (months) since the first baPWV measurement. **(C)** Four sub-groups stratified by combination of Nocturia ≥3x/night and baPWV >1800 cm/sec across the follow-up time (months) since the first baPWV measurement (ie nocturia ≤ 2x/night + baPWV ≤ 1800cm/sec; nocturia≥3x/night + baPWV ≤ 1800cm/sec; nocturia ≤ 2x/night + baPWV>1800cm/sec; nocturia≥3x/night + baPWV>1800cm/sec).

**Figure 2** illustrated the Kaplan-Meier curves for MACE-free survival according to nocturia (≤2x/night vs ≥3x/night) (**Figure 2A**), baPWV (≤1800cm/sec vs >1800cm/sec) (**Figure 2B**) and their co-existence (**Figure 2C**). Nocturia≥3x/night (HR:4.16, 95%CI:1.56-11.10), baPWV>1800 cm/sec (HR:5.07, 95%CI:1.90-13.51) and the co-existence of “nocturia≥3x/night and baPWV>1800 cm/sec” (HR:10.84, 95%CI:2.50-46.93) were significantly associated with higher incidence of MACE.

Using the -2-log likelihood value to select the model with the best performance, the addition of nocturia≥3x/night and baPWV>1800 cm/sec to the basic model (Model 1, the independent variables were age≥70, BMI≥25, waist circumference>89cm, smoker, IIEF-5≥8, IPSS≥20, HT, DM, prior history of CVA, IHD, total CVD scores>15) had the best performance in predicting MACE (difference in -2 log likelihood value: 11.219, p<0.001) (**Supplementary Table 2**).

## 4 Discussion

In the current study cohort, ~63% of the subjects had moderate to severe mLUTS (ie IPSS ≥8), whereas the global prevalence of such severity of mLUTS of similar age group is ~36% (14). As to nocturia, 98% subjects had nocturia ≥ 1x/night, 78% for ≥2x/night and ~51% for ≥ 3x/night. For comparison, the local prevalence of nocturia of similar age group in men is 63%, 49.5% for ≥2x/night and 17% for ≥3x/night, respectively (15). Earlier population-based studies showed that the figures were 68.9%-93% for ≥1x/night and 29%-59% for ≥2x/night, respectively (16). This implied that our cohort had more severe mLUTS/nocturia at baseline and they might have been more inclined to have MACE, as reported by Gacci (4)

The terminology of nocturia was first defined in 2002 [wake up at night to void] (9), revised in 2010 [each void is preceded and followed by sleep] (17) and further updated in 2018 [woken up at night, followed by sleep/intention to sleep and need of bladder diary] (18). There are advocates for using a validated 3-day bladder diary for better quantification of nocturia (19). However, a multi-centre study conducted across the UK showed that the rate of complete record of validated bladder diary is as low as 25% (20). That will cast doubt on the correct estimation of nocturia episodes of the subjects based on bladder diary in daily clinical practice. In view of the current study design, we used the definition of nocturia published in 2002, which is still widely adopted in clinical practice for its ease of use and understanding by the patients and clinicians.

Brachial-ankle PWV (baPWV) is highly correlated with aortic PWV yet its measurement is simpler and technically less demanding to quantify CAS (21), which is increasingly recognized to have preceded the development of HT (5) and served as an independent cardiovascular risk factor that can predict CAD (7), diastolic dysfunction heart failure, evolution of stroke, cardiovascular mortality and increased all-cause mortality (2, 7).

The baPWV of our cohort (1820cm/sec) was higher than the previously reported value of the general population of similar age structure (~1650cm/sec) by ~10% (12), indicating that vasculopathy appeared to be more extensive (baPWV>1800cm/sec implying risky CAS) in men with mLUTS/nocturia.

Looking closer, among the constellations of mLUTS/nocturia symptoms, independent association is only seen between baPWV and the degree of nocturia in our cohort. Similar observation was reported by Tsujimura (22). Since 30%-60% and 20-30% of men ≥60yo reported nocturia≥2x/night and ≥3x/night respectively (23), it follows that a large proportion of men presented to urology clinics for mLUTS/nocturia are at higher risk of developing MACE as compared to the general population. However, our study showed that this association is more pronounced in men<70yo. This raised the notion that younger men with more severe nocturia may have acquired more accelerated aging in their vasculature. This finding was compatible with previous observations that nocturia was associated with a 28% excess mortality risk per year (24) and that mortality was increased with the severity of nocturia in those ≤65yo but with attenuated associations in the ≥65yo (25). The potential underlying mechanisms include sleep disruption and subsequent development of related comorbidities (26). Meta-analysis suggests that individuals who had nocturia ≥3x/night contribute to a high all-cause mortality (HR:1.54, 95%CI:1.25-1.71), even after adjusting for potential confounding factors (27). We suggested this association might be mediated by the increased CAS (higher baPWV value).

Our findings also infer that men <70yo with nocturia≥3x/night may already have had CAS similar to that of the men who are 10 years older. This suggests that their arteries are “aging” faster than expected due to the process of systemic vasculopathy (e.g., atherosclerosis) other than solely an ageing (arteriosclerosis) process. This degree of CAS has likely prognosticated them to develop MACE sooner than the men of similar age in the general population. These results suggest that the degree of nocturia in men, particularly for those <70yo, may serve as an additional indicator for their vascular health, apart from demographic data such as age and history of DM/HT.

Previous study suggested that subjects with a 20% increase in baPWV (reference<1400cm/sec) was associated with a 1.3-fold increase in the risk of cardiovascular events (28). Likewise, Tomiyama reported that baPWV>1800 cm/sec implicated a hazard ratio 9.22 of developing MACE (13), in contrast to 4.56 in our cohort. 46% subjects in our cohort had baPWV >1800 cm/sec, 51% nocturia≥3x/night and ~28% subjects harbored both risk factors. In this context, the addition of LUTS in particular nocturia to the parameter baPWV may potentially reclassify the risk of developing MACE more distinctively, on top of using conventional risk factors e.g., HT, DM etc. Indeed, we also showed that nocturia≥3x/night would independently give a hazard ratio of 2.87 to future development of MACE. As the mean baPWV of men<70yo with nocturia≥3x/night nearly reached 1800 cm/sec, the cardiovascular risk of this group of men should be considered during each individualized evaluation and treatment for nocturia.

There are some proposed yet to be proven mechanisms linking nocturia and CAS to MACE. On one hand, nocturia is believed to be a determinant of non-dipping nocturnal BP (defined as reduction of nocturnal BP <10% of the daytime BP) (3), which may be associated with elevated nocturnal glomerular filtration rate(GFR) and increased CAS as determined by the PWV (29). This non-dipping effect can render hypertensive individuals prone to have more target organ damage and sooner development of MACE (30). On the other hand, LUTS is associated with hypo-perfusion of the bladder necks (31), which was inversely associated with arterial stiffness, diminished bladder compliance and lower bladder capacity (32) that led to nocturia other than global urine production, nocturnal polyuria or their combinations (33). Furthermore, 58–88% of male nocturia is associated with nocturnal polyuria (**NP**) (34), which may be associated with higher nocturnal/diurnal ratio of urinary sodium excretion (nocturnal natriuresis) as reported by Fujii (35) and raised blood pressure, which is termed “pressure-natriuresis”- in connection with stroke, left ventricular hypertrophy and congestive heart failure (36).

There are some limitations of the study: The duration of the nocturia is not taken into account for the analysis. In view of the small sample size and the single-centre design, this study was under-powered to show the association between nocturia and baPWV in men of more advanced age, for which the association was significantly more pronoucnced in the younger group of men. The follow-up time of the current study is still a bit short for the occurrence of MACE, in particular the bearing of nocturia episodes and baPWV on individual component of MACE awaits further longer observation and larger multi-centres study. Besides, the questionnaire-based self-reported of nocturia precluded studying the impact of nocturnal polyuria, nocturnal sodium excretion and the effect of functional bladder capacity, particularly low bladder capacity, on baPWV and later development of MACE. Prostate volume by transrectal ultrasound was not available in all subjects and hence its on nocturia was not evaluated. Besides, we were not able to determine if treatment of nocturia can reverse the risky CAS, or vice versa, particularly in men <70yo so as to reduce the development of MACE.

As to future research direction, it is advisable to study the relationship between baPWV, ratio of night-time to 24-hour urine production, the urinary excretion of sodium and the GFR across bedtime. Together with automated nocturnal BP for identifying dipper and non-dippers and evaluation for the sleep quality, these additional information can help to investigate the significance of nocturnal natriuresis and pressure natriuresis linking baPWV to nocturia. Besides, further longitudinal studies can be performed to determine the cause and effect relationship between nocturia and elevated baPWV; and any reversibility of the nocturia following treatment targeting at central arterial stiffness like angiotensin converting enzyme inhibitors [ACEI] (e.g. perindopril) with or without diuretics (37), new beta blockers with additional vasodilating properties on enhancing endothelial function e.g. nebivolol (38).

In conclusion, men visiting the urology clinic for mLUTS/nocturia showed a predilection for developing MACE. CAS (vascular aging), as measured by baPWV, was correlated positively with the degree of the nocturia in men, particularly when they were younger than 70yo. Nocturia≥3x/night in men was linked to risky level of CAS (baPWV>1800cm/sec), predisposing to later MACE. These two parameters may be useful in stratifying the cardiovascular risks and predicting future MACE.

## Data availability statement

The raw data supporting the conclusions of this article will be made available by the authors, without undue reservation.

## Ethics statement

The studies involving human participants were reviewed and approved by the Joint CUHK-NTEC clinical research ethics committee. The patients/participants provided their written informed consent to participate in this study.

## Author contributions

Conceptualization, investigation, methodology by CC and CN. Data curation and analysis by SY, CY, JT, PC and SWK. Funding acquisition by CN. Writing - original draft by CC and SY. Writing - review and editing by CC, CN, SY, CY, JT, PC, BL and SWK. And supervision by CN. All authors contributed to the article and approved the submitted version.
