# Supplementary figures and images for: Nocturia is associated with stiffer central artery and more likely development of major adverse cardiovascular events in men
Source: Front Urol. 2023 Jan 26;3:1113054. doi: 10.3389/fruro.2023.1113054 (PMC12327344; doi:10.3389/fruro.2023.1113054)

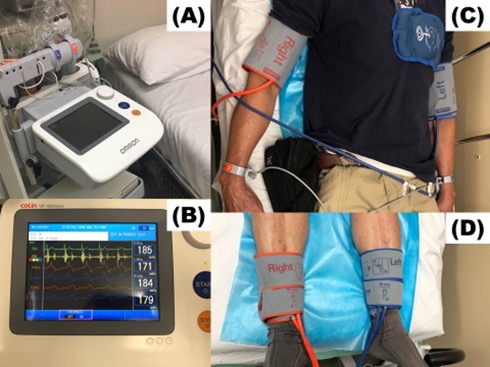

Supplement: Supplementary file 1 [file Image_1.jpeg]

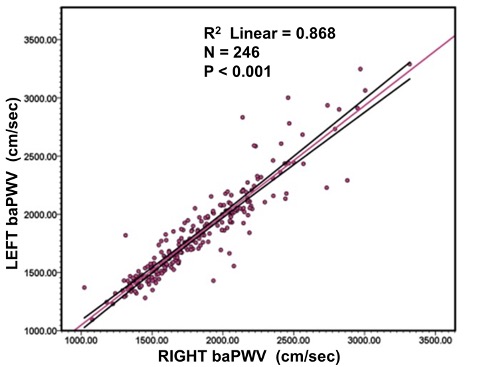

Supplement: Supplementary file 2 [file Image_2.jpeg]

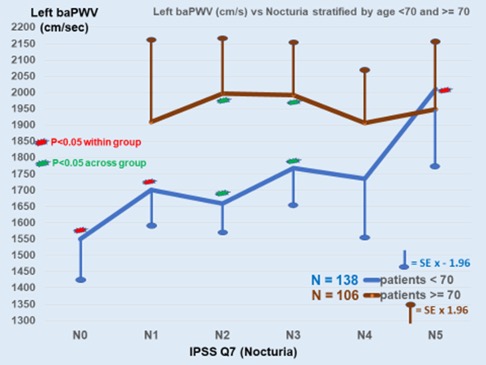

Supplement: Supplementary file 3 [file Image_3.jpeg]
