# Supplementary material for: Nocturia is associated with stiffer central artery and more likely development of major adverse cardiovascular events in men
Source: Front Urol. 2023 Jan 26;3:1113054. doi: 10.3389/fruro.2023.1113054 (PMC12327344; doi:10.3389/fruro.2023.1113054)
Supplement: Supplementary file 4 [file Table_1.docx]

Supplementary table (1)

The heart age / vascular age of the whole cohort and that stratified by each category of the groupings based on the Cardiovascular Disease Prediction Score (CVD score) proposed by D’Agostino [10]

| **Cohort / subgroup** | **Chronological age at baseline** | **Nocturia episodes at baseline** | **Ideal CVD score of men of the same chronological age without risk factors** | **Actual CVD score of the cohort / subgroup at baseline** | **Heart age / Vascular age based on CVD score at baseline** |
| --- | --- | --- | --- | --- | --- |
| **Overall** | **68.13** | **2.65** | **14** | **15.66** | **74-75** |
| **Age <70 ; nocturia ≤2** | **61.35** | **1.47** | **12** | **13.86** | **66-67** |
| **Age <70 ; nocturia ≥3** | **63.45** | **3.55** | **13** | **14.65** | **70-71** |
| **Age ≥70 ; nocturia ≤2** | **74.45** | **1.61** | **15** | **17.82** | **> 80** |
| **Age ≥70 ; nocturia ≥3** | **76.41** | **3.91** | **16** | **17.34** | **> 80** |
