# Supplementary material for: Nocturia is associated with stiffer central artery and more likely development of major adverse cardiovascular events in men
Source: Front Urol. 2023 Jan 26;3:1113054. doi: 10.3389/fruro.2023.1113054 (PMC12327344; doi:10.3389/fruro.2023.1113054)
Supplement: Supplementary file 5 [file Table_2.docx]

Supplementary Table (2): Cox proportional regression hazard analysis of factors predicting major cardiovascular events (MACE) (N=25)


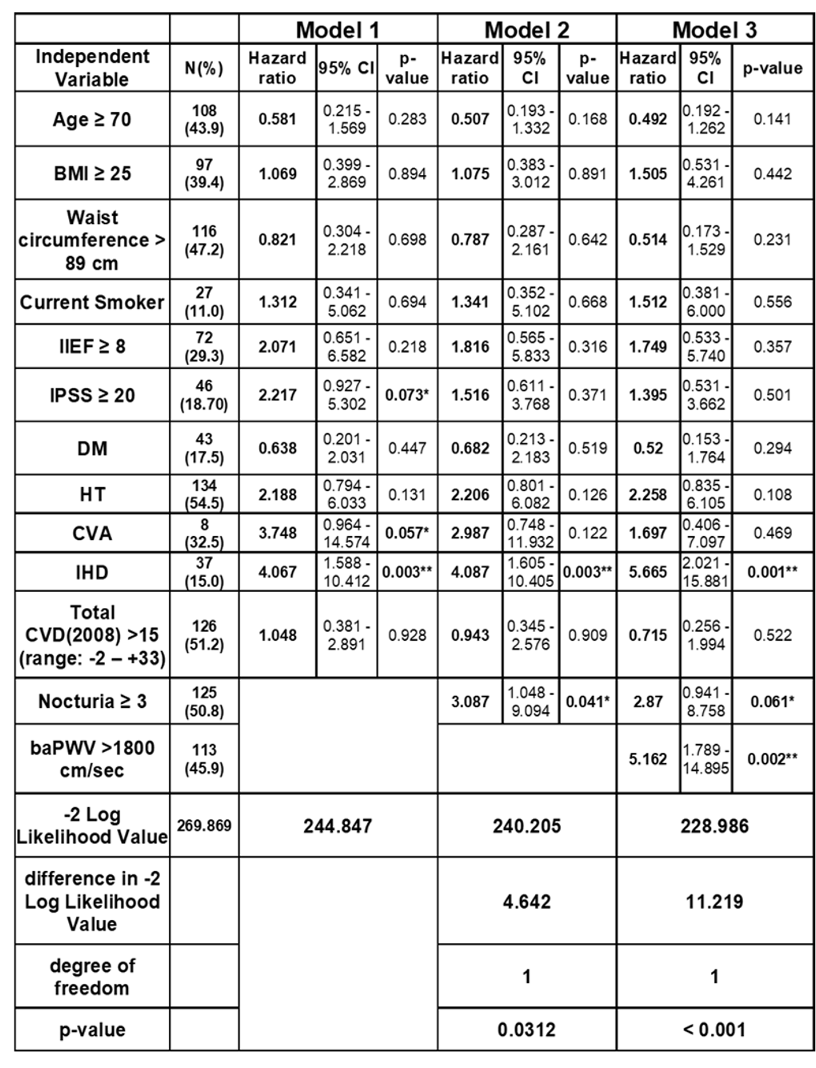


(*) = p<0.05 ; (**) = p<0.01

BMI: Body Mass Index; IIEF-5: International Index of Erectile Function; IPSS: International Prostate Symptom Score; Nocturia: Question 7 of IPSS (Nocturia); DM: Diabetes Mellitus; HT: Hypertension; CVA: History of Cerebrovascular Accident / Stroke; IHD: History of Ischemic Heart Disease; Total CVD (2008): Cardiovascular Disease Prediction Score (2008) – a composite score (range: -2 to +33) taking into account the Age / High Density Lipoprotein (HDL) level / Total Cholesterol (TC) level / Systolic Blood Pressure (sBP) / Smoking Status / History of Diabetes Mellitus (DM) [10] ; baPWV: Brachial-Ankle Pulse Wave Velocity
